# A Novel Nodule Edge Sharpness Radiomic Biomarker Improves Performance of Lung-RADS for Distinguishing Adenocarcinomas from Granulomas on Non-Contrast CT Scans

**DOI:** 10.3390/cancers13112781

**Published:** 2021-06-03

**Authors:** Mehdi Alilou, Prateek Prasanna, Kaustav Bera, Amit Gupta, Prabhakar Rajiah, Michael Yang, Frank Jacono, Vamsidhar Velcheti, Robert Gilkeson, Philip Linden, Anant Madabhushi

**Affiliations:** 1 Department of Biomedical Engineering, Case Western Reserve University, Cleveland, OH 44106, USA; kxb413@case.edu (K.B.); axm788@case.edu (A.M.); 2 Department of Biomedical Informatics, Stony Brook University, Stony Brook, NY 11794, USA; prateek.prasanna@stonybrook.edu; 3 University Hospitals Cleveland Medical Center, Case Western Reserve University, Cleveland, OH 44106, USA; amit.gupta@uhhospitals.org (A.G.); Michael.Yang@uhhospitals.org (M.Y.); fjj@case.edu (F.J.); Robert.Gilkeson@uhhospitals.org (R.G.); Philip.Linden@uhhospitals.org (P.L.); 4 Department of Radiology, Mayo Clinic, Rochester, MN 55970, USA; radpr73@gmail.com; 5 Laura and Isaac Perlmutter Cancer Center, NYU Langone, New York, NY 10016, USA; Vamsidhar.Velcheti@nyulangone.org; 6 Louis Stokes Cleveland Veterans Administration Medical Center, Cleveland, OH 44106, USA

**Keywords:** lung nodule classification, CT images, lung-RADS, nodule interface sharpness, nodule risk score

## Abstract

**Simple Summary:**

The great majority of pulmonary nodules on screening CT scans are benign (95%). Due to inaccurate diagnoses of granulomas from adenocarcinomas on CT scans, many patients with benign nodules are subjected to unnecessary surgical procedures. The aim of this retrospective study is to evaluate the discriminability of a new radiomic feature, nodule edge/interface sharpness (NIS), for distinguishing lung adenocarcinomas from benign granulomas on non-contrast CT scans. Moreover, we aim to evaluate whether NIS can improve the performance of Lung-RADS, by reclassifying benign nodules that were initially assessed as suspicious. In a cohort of 352 patients with diagnostic non-contrast CT scans, NIS radiomics was able to classify nodules with an area under the receiver operating characteristic curve (ROC AUC) of 0.77, and when combined with intra-tumoral textural and shape features, classification performance increased to AUC of 0.84. Additionally, the NIS classifier correctly reclassified 46% of those lesions that were actually benign but deemed suspicious by Lung-RADS. Combining NIS with Lung-RADS has the potential to alter patient management by significantly decreasing unnecessary biopsies/follow up imaging.

**Abstract:**

The aim of this study is to evaluate whether NIS radiomics can distinguish lung adenocarcinomas from granulomas on non-contrast CT scans, and also to improve the performance of Lung-RADS by reclassifying benign nodules that were initially assessed as suspicious. The screening or standard diagnostic non-contrast CT scans of 362 patients was divided into training (S_t_, *N* = 145), validation (S_v_, *N* = 145), and independent validation (S_iv_, *N* = 62) sets from different institutions. Nodules were identified and manually segmented on CT images by a radiologist. A series of 264 features relating to the edge sharpness transition from the inside to the outside of the nodule were extracted. The top 10 features were used to train a linear discriminant analysis (LDA) machine learning classifier on St. In conjunction with the LDA classifier, NIS radiomics classified nodules with an AUC of 0.82 ± 0.04, 0.77, and 0.71 respectively on S_t_, S_v_, and S_iv_. We evaluated the ability of the NIS classifier to determine the proportion of the patients in S_v_ that were identified initially as suspicious by Lung-RADS but were reclassified as benign by applying the NIS scores. The NIS classifier was able to correctly reclassify 46% of those lesions that were actually benign but deemed suspicious by Lung-RADS alone on S_v_.

## 1. Introduction

The great majority of pulmonary nodules on screening CT scans are benign (95%), and a number of benign nodules tend to be granulomas [1]. On the other hand, adenocarcinoma is considered as the most common form of lung cancer, accounting for about 40% of all non-small cell lung cancer (NSCLC) occurrences. Due to their similarity in appearance to adenocarcinomas, granulomas are considered among the most difficult tumor confounders to discern on CT scans [2,3]. Differentiating granulomas and adenocarcinomas can be challenging to diagnose on positron emission tomography (PET) scans as well, both appearing “hot” on PET. On account of the inability to distinguish these lesions on CT scans, many patients with benign nodules are subjected to unnecessary surgical procedures and harmful radiation [4]. Considering the benefit of CT scans for lung cancer screening, the American College of Radiology (ACR) introduced the Lung Imaging Reporting and Data System (Lung-RADS), a classification system designed to standardize lung reporting screening examinations [5]. According to Lung-RADS criteria [6], the likelihood of a nodule being malignant is characterized based on the nodule size. Lung-RADS employs a risk scale from 0 to 4, with 0 suggesting a benign and 4 suggesting the presence of a malignant nodule [7]. However, Lung-RADS can produce many false positive errors, especially concerning nodules with an average diameter greater than 8 mm, which could be assigned to either of the 4A or 4B categories.

Computer-aided diagnostic (CADx) tools [8] aim to maximize cancer detection sensitivity and specificity, while also attempting to minimize the interpretation time for radiologists [9,10,11,12]. CADx tools are often driven by “radiomics” [13], a term referring to high throughput computerized extraction of image features for improved nodule characterization on CT scans. Most radiomic-based approaches involve shape [12,14] or texture-based characterization of the nodule, the goal being to identify a set of shape and texture features that can distinguish benign from malignant nodules ([15], p. 1). Textural feature extraction allows for describing the spatial arrangement of intensities and heterogeneity patterns of tumor phenotypes on radiographic scans. On the other hand, shape-based features tend to capture the irregularities along the nodule surface, which in turn may be a reflection of internal heterogeneity and may represent differences in growth patterns. The majority of radiomic approaches in lung cancer have been mainly focused on intra-tumoral textural analysis [16,17,18]. However, there is increasing evidence that heterogeneity patterns associated with the peri-tumoral region, the area immediately surrounding the tumor, might present informative diagnostic and prognostic cues. For instance, in [19], the authors showed that immune response signatures such as the presence of peri-tumoral lymphocytes are associated with disease-specific survival. A recent study [20] showed that the combination of intra-tumoral and peri-tumoral radiomic features can yield to a better discrimination of lung nodules compared to intra-tumoral texture features alone on screening CT scans.

Another class of approaches are deep learning (DL) models [21]. DL models have been proposed for automatically learning the most discriminating features for distinguishing benign from malignant nodules on CT scans [22,23]. Multiple papers [24,25,26] have already explored the potential of deep networks for detecting and segmenting the pulmonary nodules on CT scans [27], and there is a growing interest in the use of these models for diagnosis and classification of lung nodules on CT scans [22,23,28]. However, these approaches have mostly been employed on non-granulomatous benign lesions which seems to be an easier task than resolving granulomas from adenocarcinomas.

**Figure 1:**
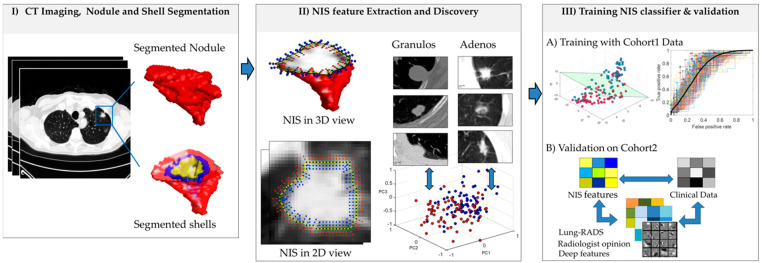
Illustrative flowchart depicting the NIS approach.

Recently there has been a growing appreciation of the role of lymphocytic infiltration associated with malignant lung nodules [29]. The infiltration appears to be localized within the peri-nodular space of malignant nodules, which may explain differential textural patterns adjacent to the nodule. In this work, we present a new radiomics approach called nodule interface sharpness for characterization of lung nodules on CT scans. The main hypothesis of this approach is that the adenocarcinomas and granulomas present with different lymphocytic infiltration patterns, and NIS radiomics is able to capture these differences in the form of transitional heterogeneity-related features from the intra- to the peri- nodular space. Hence, by capturing and characterizing the transitional heterogeneity from the intra- to the peri- nodular space, we will be able to distinguish granulomas from adenocarcinomas. In this study, we evaluate the utility of NIS in distinguishing between granulomas and adenocarcinomas on routine lung CT scans. Figure 1 represents the methodological pipeline for the NIS classifier construction and evaluation. A total of 362 patients comprising an equal number of granulomas and adenocarcinomas were considered in this study, with the dataset randomly and equally divided into training (S_t_, *N* = 145), validation (S_v_, *N* = 145) and independent validation (S_iv_, *N* = 62) sets. The performance of the NIS classifier was assessed based off the area under the receiver operating characteristic curve and compared with the performance of (a) intra-tumoral texture, (b) peri-tumoral texture, and (c) deep learning features. In this study, we also sought to evaluate whether NIS radiomic features improve the performance of Lung-RADS by reducing the number of cases that are actually benign and categorized as suspicious by lung-RADS criteria.

## 2. Materials and Methods

Our study was Health Insurance Portability and Accountability Act (HIPAA) compliant and institutional review board (IRB) approved. This research has been approved by University Hospitals IRB (ethics committee) on 25 June 2019 (ethics code: STUDY20190887). A retrospective chart review with de-identified data was employed and no PHIs were used. Thus, the need for informed consent from all patients was waived.

**Figure 2:**
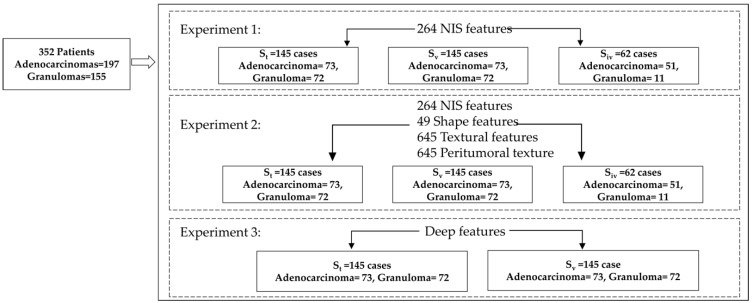
The dataset inclusion and experimental design of the study.

Data set: This retrospective study comprised of CT scans of 362 patients from multiple institutions. The data set of 362 patients was divided equally into training (S_t_, *N* = 145) and validation (S_v_, *N* = 145) and independent validation (S_iv_, *N* = 62) sets. Between 1 January 2007 and 31 December 2020, radiology image archives of participating institutions were searched consecutively to identify 471 patients who either had a granuloma or an adenocarcinoma as confirmed via histopathology. Patients who met the following criteria were included: (a) availability of pathology report via surgical wedge resection/biospy, (b) presence of a screening or diagnostic thoracic CT scan in axial view, and (c) presence of a solitary pulmonary nodule. To this cohort of 405 patients, we applied the exclusion criteria of removing scans with CT artifacts (*n* = 48), presence of imaging contrast (*n* = 37) and patients who underwent biopsy prior to imaging (*n* = 30). The final cohort had 290 patients (Figure 2), which were divided into a training set (S_t_) that consisted of 145 patients with 73 adenocarcinomas and 72 granulomas, and a test set (S_v_) that contained 73 adenocarcinomas and 72 granulomas. Additionally, a set of 62 cases (S_iv_) from an independent institurion including 11 granulomas and 51 adenocarcinomas was included for further independent validation of the NIS features.

The CT scan images were acquired from either Siemens (Sygno, Siemens AG, Erlangen, Germany), General Electric (Lightspeed16, GE Medical Systems, Waukesha, WI, USA), Philips (iCT, Philips Medical Systems, Cleveland, OH, USA), or Toshiba (Aquilion, Tochigi-ken, Japan) CT systems. A subset of these data has been previously published [14,30,31,32], where intra-tumoral texture, nodule shape and vessel tortuosity features were evaluated in terms of their ability to distinguish granulomas from adenocarcinomas. This dataset was also previously used [20] to study the potential of peri-tumoral texture features in distinguishing adenocarcinomas and granulomas.

**Figure 3:**
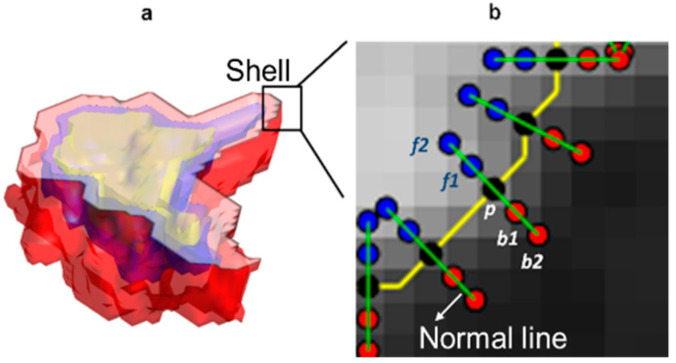
A pictorial representation of the NIS feature extraction. (**a**) A nodule is partitioned into three nested shells (denoted by yellow, purple and pink colors). The annular shells are computed by applying binary dilation and erosion on the nodules volume. (**b**) 2D representation of the outer shell’s border pixels (black dots) and their corresponding normal lines (green lines) including inner (${\;f}_{i}$, blue dots) and outer (${\;b}_{i}$, red dots) pixels over the normal line. The yellow line denotes the boundary of the shell.

Nodule Segmentation and Feature Extraction: The nodules were identified by a board-certified cardiothoracic radiologist with 20 years of experience, and the region of interest (ROI) was manually segmented across all the 2D slices of the nodule via a hand-annotation tool in axial view using an open source software (3D Slicer 4.7) [33]. The radiologist was blinded to the pathologic diagnosis, but in order to efficiently annotate the nodule, clinical information such as age was provided to the reader. Additionally, the radiologist was given the option to vary the window and level setting within this software. Following manual annotation by the radiologist, post-processing was performed and the segmented nodule volume was automatically partitioned into three nested shells (Figure 3a) including inner, *Sh_i_*, middle *Sh_m_*, and the outer *Sh_o_* shells. Each shell is comprised of multiple 2D slices in the Z direction, which in turn is comprised of a set of boundary pixels. As illustrated in Figure 3b, for a boundary pixel *p*, the slope of the normal line was computed using the co-ordinates of the pixel *p* and its immediate adjacent pixels over the boundary. The normal line at boundary pixel (*p*) is then sampled into inner (fi) and outer (bi) pixels (as presented by red and blue dots on Figure 3b) in which inner pixels lie inside the boundary of a specific shell while outer pixels lie outside the shell in the peri-tumoral space.

The heterogeneity transition and margin sharpness were then computed for the shells by considering the grayscale intensity profile and its corresponding gradient magnitude along the normal lines at each border pixel (*p*). For each boundary pixel *p*, 10 core NIS features were computed over the perpendicular line of *p,* denoted as *l_p_*. The core features included: the average grayscale intensity differences between the inner and outer pixels over *l_p_*, statistics of the grayscale intensity profiles, as well as the derivate of the grayscale intensity profiles over *l_p_* and point to point grayscale intensity difference over *l_p_*. These core features then generalized to higher level features to describe the slices, shells, and the entire nodule by taking the first order and second order statistics of the lower level core features.

A set of 88 features was computed per nodule shelling. As three shells were considered per nodule, a total of 264 NIS features were computed per nodule. All feature values were normalized (mean of 0 and a standard deviation of 1). A pictorial representation of the process of NIS feature extraction is illustrated in Figure 3. Additional details on the mathematical description of core NIS features are presented in Appendix A.1. 

Statistical Analysis: Statistical analysis was performed on MATLAB 2018b platform (Mathworks Inc, Natick, MA, USA). To avoid the curse of dimensionality and reduce the risk of overfitting, Wilcoxon rank sum test was implemented as a feature selection method was employed to identify only the top discriminating NIS features with the lowest unadjusted *p*-value (*p* < 0.05). One specific attribute desirable in radiomic features is that the feature expression should minimally change for test-retest scans acquired within a short interval [34]. To assess the stability of the NIS features, we used the independent reference imaging database to evaluate response (RIDER) [35] lung cancer dataset which consists of same-day repeated test and re-test CT scans for 31 patients. The intra-class correlation coefficient (ICC) was used to assess the stability of the top NIS features identified in feature selection process. ICC varies between −1 and 1, where ICC = 1 corresponds to a highly reproducible feature and ICC = 0 corresponds to a feature which is not highly reproducible and hence unstable.

The most stable + discriminating features were selected by identifying the most discriminating features that had an inter-correlation coefficient (ICC) > 0.9. The top ten stable + discriminative features used for further evaluation in conjunction with the machine classifiers using S_t_ [9]. These features were evaluated in conjunction with a linear discriminant analysis (LDA), support vector machines (SVM-linear and RBF kernels) [36], naïve Bayes, and K-nearest neighbor (KNN) classifiers. The AUC cross validation performance on S_t_ was the criterion chosen for identifying the most accurate classifier.

Experimental Design:

Experiment 1: Supervised classification and unsupervised clustering of the nodules by NIS features.

In this experiment, first, we evaluated the performance of the machine classifiers on S_v._ Then, hierarchical unsupervised clustering was performed on nodules with the most informative NIS features. The goal here was to evaluate how the concordance between the dominant nodule clusters and the histopathologic labels (i.e., adenocarcinoma or granuloma). We also sought to evaluate and contrast the visual differences between the emerging nodule clusters. Lastly, we evaluated how the NIS radiomic features vary as a function of different CT image acquisition parameters, including manufacturer, slice thickness, and type of scan. We also evaluated the sensitivity of NIS-based classifier (M_NIS_) as a function of CT slice thickness.

Experiment 2: Comparative strategies of NIS with radiomic features, deep models, and human readers in discriminating adenocarcinomas from granulomas.

2A. Comparison with other radiomic feature families: We sought to compare M_NIS_ (i.e., the classifier trained with NIS features) against three radiomic-based classifiers, including a shape-based classifier (M_Shape_) trained with 49 2D and 3D nodule shape features as well as two texture -based classifiers (M_Peri-Tex_ and M_Intra-Tex_) trained with 516 texture features from intra-nodular and peri-nodular regions. These features have been previously shown to have utility in distinguishing between malignant and benign nodules on non-contrast CT scans [16,20]. The texture features included Haralick [37], wavelet-based Gabor [38] responses and Laws [39]. M_NIS_, M_Shape_, M_Intra-Tex_ and M_Peri-Tex_ were trained on S_t_ and evaluated on S_v_ for their ability to distinguish adenocarcinomas and granulomas in terms of ROC AUC. Additionally, we trained a combined classifier M_All_ using the top informative NIS, shape, intra- and peri-tumoral texture features on S_t_ and validated M_All_ on S_v_. The LDA algorithm was used for the combined classification strategy. The top five features of each feature group were initially selected on S_t_ and consequently M_All_ was trained with the 20 concatenated features, in turn comprising the top five features from each feature group.

2B. Comparison with deep networks: The performance of M_NIS_ was compared against deep learning classifiers. Two deep networks, including a simple 2D LeNet [40] architecture, a 3D CNN, were used for this purpose. Additionally, the attention maps [41] for the deep networks were calculated to determine the specific spatial locations on the nodules where the CNN appears to focus its attention in order to best distinguish the nodules in S_t_. The LeNet and 3D CNN architectures comprised two sets of convolutional, rectified linear unit (ReLU) activation and pooling layers, followed by a fully-connected layer, activation, another fully-connected, and finally a softmax classifier. The learned weights were then evaluated on S_v_, and the predicted probabilities were utilized to generate the ROC curve. To extract deep features using a 2D CNN, 2D patches with a receptive field size of 80 × 80 pixels cropped at the center of the nodule across all slices were obtained and then inputted to a 2D CNN. The 3D model also used 3D patches with a receptive field size of 50 pixels in the XY plane and 10 slices in the Z plane (50 × 50 × 10).

2C. Comparison with human readers: M_NIS_ was compared against the interpretations of two human readers for the nodules in S_v_. Reader 1 (R1) was a board-certified attending radiologist with 11 years of experience in thoracic radiology, and Reader 2 (R2) was a pulmonologist with three years of experience in reading chest CT scans. The readers scored the nodules between 1–5 (Score 1 ‘benign’, score 2 ‘probably benign’, score 3 ‘indeterminate’, score 4 ‘probably malignant’ and score 5 ‘malignant’) according to the rules we already applied in [32]. In this study, a reader’s score of 4–5 is considered to be equivalent with Lung-RADS 4A and 4B. We also computed the agreement between the M_NIS_ and R1 and R2 for differentiating both adenocarcinomas and granulomas.

Experiment 3: Assessing ability of M_NIS_ to reclassify lesions originally classified as suspicious by Lung-RADS: 

In this experiment, the nodules were scored under supervision of collaborating radiologist by Lung-RADS, Version 1.1 criteria. In this regard, the average of major and minor diameters for all of the 145 cases in S_v_ was measured by the radiologist using the 3D slicer. Accordingly, nodules were assigned to risk categories based on lung-RADS. To determine the proportion of patients who could have possibly avoided a biopsy or intervention via the use of the NIS classifier over Lung-RADS, we defined biopsy reduction benefit of NIS (B_NIS_). N_b_ was used to denote the number of patients identified as suspicious according to Lung-RADS. A proportion of N_b_ cases being evaluated as benign by NIS classifier were labelled as being down-graded N_d_. Based on the ground truth histopathology report, N_d_ cases were further categorized into truly downgraded cases, which were benign as per pathology (N_T,d_), and incorrectly downgraded cases, which were originally malignant as per pathology (N_F,d_). B_NIS_ was calculated as the ratio of N_T,d_ to N_b_.

## 3. Results

**Figure 4:**
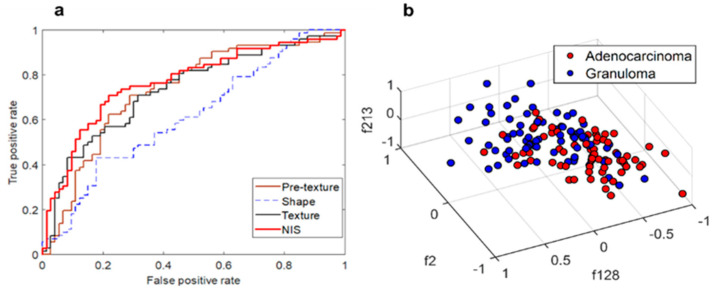
The performance of NIS features in supervised and unsupervised learning settings. (**a**) ROC curve of classifiers trained with NIS and other radiomic features in S_v_. (**b**) Nodules represented in the space of top 3 NIS features. As figure suggests, two classes of nodules within top 3 discriminating NIS features appear to be separable.

**Figure 5:**
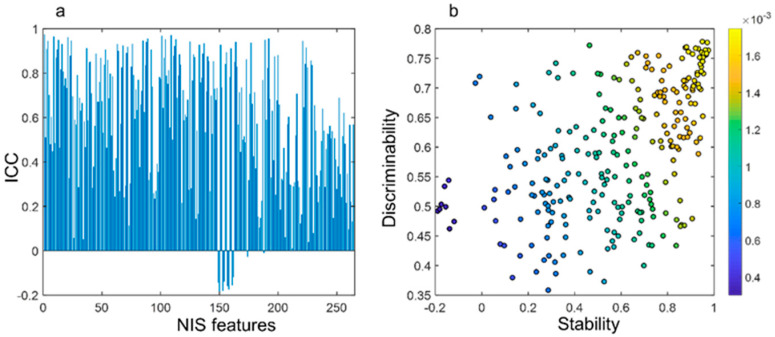
NIS stability and discriminability analysis. (**a**) Moderate to high stability of 264 NIS features with ICC > 0.6. (**b**) Feature significance as a function of stability and discriminability.

Experiment 1: Supervised classification and unsupervised clustering of the nodules by NIS features:

Supervised classification and stability analysis results: The M_NIS_ yielded an AUC of 0.82 ± 0.04 and 0.77 and 0.071 respectively in in S_t_, S_v_, and S_iv_ sets. Figure 4b represents the 3D cluster plot of adenocarcinomas (red dots) and granulomas (blue dots) within S_t_ in a 3D feature space involving the three most discriminating and stable NIS features. Among 264 features, 73 features (26%) were found to be highly reproducible (stable) with intra class correlation (ICC > 0.8) between repeated measurement of 31 test-retest scans of RIDER dataset. As shown in Figure 5a the majority of features (54%) were found either moderately or highly stable with ICC > 0.6. The top 10 features were then selected among features with very high stability (ICC > 0.9) and discriminability (AUC > 0.75).

Figure 5b illustrates the significance of NIS features as a function of their stability and discriminability. The data points scattered on up right of this figure correspond to the NIS features that are both discriminative and stable between repeated scans.

Distribution of the nodules among various CT parameters: Clinical parameters ‘Smoking status’ and ‘Age’ were the only patient factors that were found to be significantly different between the two nodule classes, adenocarcinoma and granuloma. Table A3 in Appendix A.2 provides the statistical relationship of patient characteristics and CT parameters with diagnostic class of the nodules. The influence of reconstruction kernel on CT radiomics has been demonstrated by several groups, and therefore precaution was taken to maintain a class balance of reconstruction kernels in both the S_t_ and S_v_. Furthermore, M_NIS_ (*n* = 145) was independently validated to assess the effect of slice thickness. The highest AUC of 0.78 was obtained on diagnostic scans with smaller slice thickness (≤3 mm). Additionally, we determined that there was no statistically significant association of a nodule’s spatial location with its corresponding diagnostic class. The details are provided in Appendix A.3.

**Figure 6:**
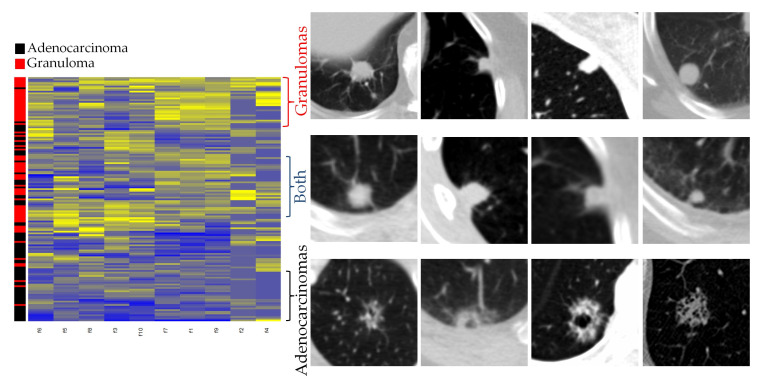
Hierarchical clustering of nodules described with top NIS features (**left panel**). (**Right panel**): Four example nodules per each emerging sub group/clusters corresponding to granulomas, suspicious nodules and adenocarcinomas.

**Figure 7:**
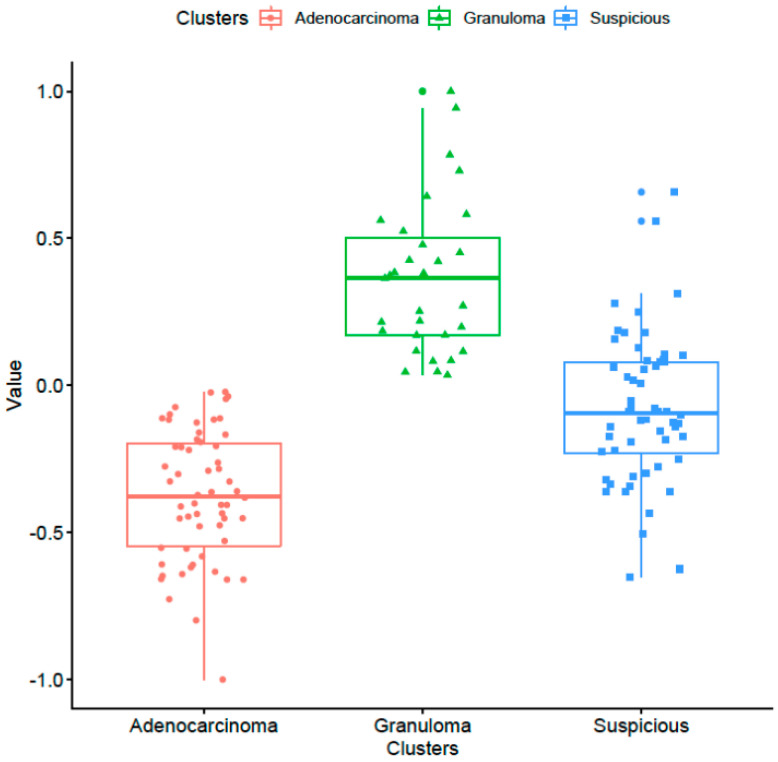
The distribution of the top NIS feature values among the dominant adenocarcinoma, granuloma and suspicious clusters that emerged via unsupervised clustering.

Unsupervised clustering analysis: Unsupervised hierarchical clustering of the nodules described into two clusters as per top 10 NIS stable + discriminating features yielded sensitivity and specificity of 0.71 and 0.66, respectively. The hierarchical clustering analysis (heat map in Figure 6, left panel) revealed three dominant groupings of nodules. These three groupings of the clustered patients when compared to the true pathological results can be visually divided into (a) mostly granulomas, (b) a combination of adenocarcinomas and granulomas (suspicious nodules) and (c) mostly adenocarcinomas groups. As is clear from the heat map shown in Figure 6 and the corresponding nodule exemplars of each of these three clusters (right panel), granulomas tend to have higher NIS values (i.e., sharper boundary) while the suspicious nodules group that consisted of both granulomas and adenocarcinomas tend to have moderate NIS values, and the cluster corresponding to adenocarcinomas have lowest NIS values. Figure 7 shows the distribution of the top NIS feature values among the three categories that emerged as a result of unsupervised clustering.

Experiment 2: Comparative strategies of NIS with radiomic features, deep models, and human readers in discriminating adenocarcinomas from granulomas.

2A. Comparison results with radiomics: As shown in Table 1 and Figure 4a, M_NIS_ outperformed the classifiers trained with well-known radiomic features in S_v_ and S_iv_. The AUC between M_NIS_ and other classifiers was found to be statistically different in S_v_ (p < 0.05). Additionally, combining NIS with other radiomic features resulted in a classifier M_All_ that yielded an AUC of 0.91 ± 0.03 and 0.80, 0.70 in S_t_, S_v_ and S_iv_ respectively. The accuracy of the models is provided in the Appendix A.5.

**Figure 8:**
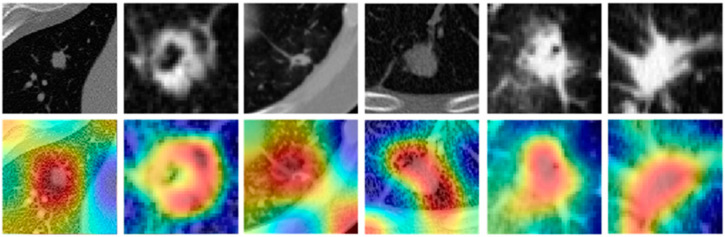
Six pulmonary nodules and the corresponding activation maps generated by CNN. CNN’s attention maps (**bottom row**) shows that the deep learning model relies not only on the nodule surface but also on the immediate periphery region to discriminate nodules. The model learns from the nodule periphery especially in the consolidated nodules (**panels 1**, **3**, **4** from **left**).

2B. Comparison results with deep learning models: The CNN models were trained with more than 100 epochs, after which the weights were locked down for testing (Appendix A.4). Weights learned from the training phase were then used on S_v_ to classify nodules. The predicted probabilities obtained by 2D CNN yielded an AUC of 0.76 while 3D CNN’s classification yielded an AUC of 0.68 on S_v_. We computed the attention maps of a 2D CNN that our group used in [32]. The attention maps were computed to identify the specific spatial regions on the nodule and surrounding region on CT scans where the CNN focused its attention in order to discriminate the nodules. Figure 8 illustrates six nodules taken with different scanning parameters and the corresponding attention maps of the deep model. As can be seen from the attention maps, the CNN appears to focus not only on the nodule surface but also on the immediate periphery of the nodules. The visual attention maps appear to provide implicit confirmation of the importance of the spatial regions within and around the nodule that the NIS radiomic feature is interrogating.

2C. Multi-reader study results: An agreement of 60% and 63%, respectively, was observed between M_NIS_ and 2 human readers in distinguishing adenocarcinomas. The machine reader agreement (MRA) was computed based on the following equation in which M_adeno_ is the set of adenocarcinomas identified by M_NIS_, and R_adeno_ is the set of adenocarcinomas identified by a reader.

(1) $$MRA=\frac{\left|M_{adeno}\cap R_{adeno}\right|}{\left|R_{adeno}\right|}$$

**Figure 9:**
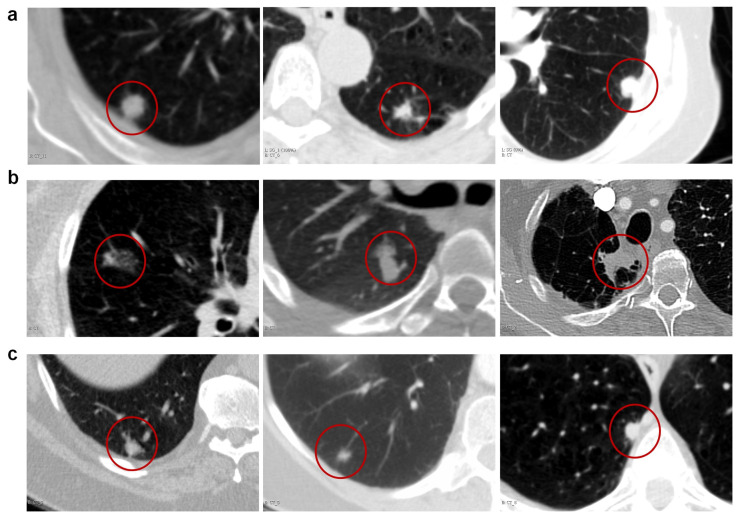
Examples of difficult cases that misclassified by readers. (**a**) Axial CT images of biopsy proven three pulmonary adenocarcinomas that were misclassified by both readers and correctly classified by machine (NIS classifier). (**b**) Three pulmonary granulomas that were misclassified by both readers and correctly classified by machine. (**c**) Three pulmonary adenocarcinomas that were misclassified by both human and machine.

MRA was found to be 56% and 64% respectively between the classifications results of M_NIS_ as compared to the two readers in distinguishing granulomas. Additionally, we identified that some of the nodules were misclassified by readers and correctly classified by NIS classifier (Figure 9). This might help to model confounder nodules for human readers. Figure 9 qualitatively illustrates some of the difficult cases that were misclassified by human readers, but correctly identified by the M_NIS_. Interestingly, checking the slice thickness of the nodules that were misclassified by M_NIS_, we noted that all three were of low resolution and had a slice thickness of greater than 4 mm, suggesting that the classifier was challenged by the large slice spacing and poor resolution and quality.

Experiment 3: Assessing ability of M_NIS_ to reclassify lesions originally classified as suspicious by Lung-RADS.

**Figure 10:**
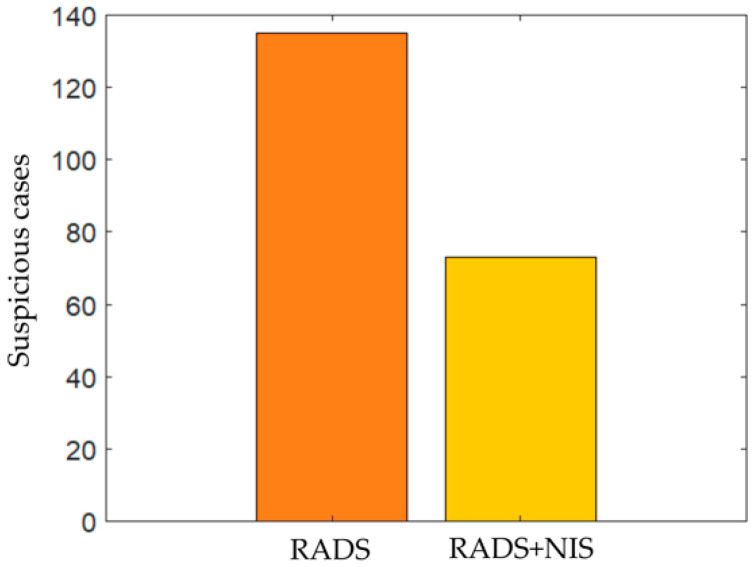
The number of suspicious cases decreased from 135 to 73 by combination Lung-RADS and M_NIS_ scores on S_v_. The 62 downgraded cases would be considered as probably benign or Lung-RADS 3 category.

By combining M_NIS_ with Lung-RADS criteria, a number of patients who were initially labelled as suspicious for malignancy by Lung-RADS alone were downgraded to probably benign (equivalent to Lung-RADS 3). The downgrade improvement was between 27% and 46%. As shown in Table 2, 135 out of 145 validation set patients were evaluated as suspicious by our radiologist (Lung-RADS 4A and 4B equivalent with reader’s score 4–5) and consequently might need either biopsy or additional imaging. However, considering the M_NIS_ benignity probability of these 135 patients, a minimum of 36 and maximum of 62 patients could be downgraded to probably benign (RADS 3) category. As shown in Figure 10, considering M_NIS_ benignity of 0.58 as a criterion of downgrading from the suspicious to probably benign category leads to 62 downgraded patients with 46 true positives and 16 false positives, yielding an overall NIS_Benefit of 0.74. Increasing the M_NIS_ benignity confidence bar to 0.98 in turn led to 36 patients being downgraded.

## 4. Discussion

There has been substantial evidence that low dose CT screening enables early detection of lung cancers and hence can be effective in decreasing the morbidity and mortality associated with the disease [42]. However, radiologist interpretation of lung CT scans is subject to inter-reader variability [43].

In this work, we presented a new radiomics approach (NIS) for lung nodule characterization and investigated the ability of NIS radiomic features extracted from periphery of the lung nodules and adjacent peri-tumoral zone on CT to distinguish adenocarcinomas from granulomas. Since the nodule boundary in malignant nodules is an active zone, NIS aimed to capture the heterogeneity and texture transition from the boundary region which starts from 2 mm inside to 2 mm outside of the nodule.

Granulomas and adenocarcinomas are the most common representation for benign and malignant lung nodules on CT scans. Distinguishing granulomas from adenocarcinomas is confounded by their similar visual appearance on routine CT scans. Unfortunately, due to this complexity, many patients with benign granulomas are subjected to unnecessary surgical resections and biopsies or additional higher dose CT tests. This suggests the need for improved computerized characterization of these nodules in order to distinguish between these two classes of lesions on CT scans. Our findings showed that M_NIS_, a machine learning classifier trained with NIS features, extracted from 2 mm inside and outside of the nodule interface could discriminate adenocarcinomas from granulomas with AUC of 0.77. Stability is a desirable attribute in radiomic features, meaning that the feature expression should either not change (or minimally change) for test-retest scans acquired within a short interval duration [34,44]. In this study, we focused on identifying and selecting those features that were not only associated with the likelihood of malignancy of a nodule, but also stable in repeat scans. The majority of NIS features (54%) were found either moderately or highly stable with ICC > 0.6 in repeat scans. We also studied the impact of slice thickness and patient parameters to the performance of the M_NIS_ classifier. Performing a slice thickness subset analysis, we found that the highest AUC was obtained on diagnostic scans with a slice thickness ≤3 mm and M_NIS_ misclassifications tended to be in those scans with slice thickness >4 mm.

It is has been previously shown [45] that during cancer invasion and its metastatic spread, peri-tumoral stroma undergoes changes. This is evidenced by an increased presence of immune cells and fibroblasts, which can help deposit extracellular matrix and reorganize the stromal network. In rapidly growing tumors, hypoxia also alters the tumor microenvironment and exerts an effect on the surrounding cells. In contrast, the periphery of a pulmonary granuloma is a completely different microenvironment [46] and there are differences based on different etiologies. Since the tumor cells are very densely packed in the center, the peri-tumoral zone represents the advancing front of the cancer (containing fewer and more sparsely distributed cells). Additionally, in [47], the authors showed that heterogeneity of tumor boundary and proximal peritumoral stroma was more useful for differentiating low-risk from non-low-risk tumors compared to the interior of the tumor or the distal peritumoral stroma. Since the tumor cells are very densely packed in the center, the peritumoral zone represents the advancing front of the cancer (containing fewer and more sparsely distributed cells).

The majority of radiomic approaches used in lung cancer have focused solely on malignant lung nodule texture analysis and shape features from non-contrast CT exams [16,17,18,48,49,50]. For instance, a study [18] used an intra-nodular radiomics based approach, using only Haralick features to distinguish adenocarcinoma from granuloma, and obtained a sensitivity of 88%. However, the dataset consisted of only 55 nodules from a single site and their model was not validated on an independent dataset. Alilou et al. [14] showed that shape based features (such as roughness, convexity, and sphericity) are able to distinguish adenocarcinomas from granulomas with an AUC of 0.72 on an independent test set of 67 patients. Our group has previously studied the performance of intra-tumoral and peri-tumoral textures in discriminating lung nodules in [20,31]. We found that, in representative H&E stained images corresponding to the resected nodule, the interface of the tumor had a ‘rim’ of increased tumor infiltrating lymphocytes (TILs) and tumor associated macrophages (TAM). At a macroscopic scale, this densely packed stromal TILs around adenocarcinomas manifest as smooth interface texture on CT and potentially results in lower expression of NIS features that accounts for a smoother transition of texture from inside to outside of adenocarcinomas compared to granulomas. On the other hand, higher NIS values in granulomas account for a sharper texture transition in the nodule interface.

In this study, we found that M_NIS_ outperformed the classification results of other shaped and textural based classifiers (M_Shape_, M_Intra-Tex_ and M_Peri-Tex_) on S_v_ nd S_iv_. The discrimination performance could be boosted by up to 0.80 when we trained a classifier with combining NIS with other intra- and peri-tumoral texture and shape radiomic features.

Recently, deep learning models [21] have been proposed for automatic learning of discriminating features from nodules regions. Multiple papers [24,25,26] have explored the potential of deep networks for the detection of pulmonary nodules and there is a growing interest in the use of deep learning models for diagnosis and classification of lung nodules on CT scans [22,23]. Most published deep models have not been evaluated on an independent validation dataset. For instance, in [51], the authors proposed a deep approach to classify malignant and benign nodules with an accuracy of 75% and sensitivity of 83% on a publicly available dataset comprising 4323 nodules in a cross validation setting. We performed attention map analysis on our 2D CNN network, as shown in Figure 8. The analysis revealed that the nodule surroundings have an important impact on the decision of the deep model. This suggests nodule interface region quantified by NIS features appears to be important for nodule classification.

The Lung-RADS tool provides five categories to differentiate high-risk from low-risk nodules as per nodule morphology, size, and growth. Its risk groups include categories 1 (negative), 2 (benign appearance), 3 (probably benign), and 4 (suspicious) [52]. Despite using lung-RADS criteria, radiologist interpretations cause a significant number of lung nodules being labelled as either indeterminate or suspicious for malignancy (false positive rate of 10.4%) [6], which in turn leads to potentially avoidable negative biopsies and/or additional radiation exposure to the patient. We showed that combining M_NIS_ with Lung-RADS criteria could reliably downgrade 27–46% of the patients who were initially labelled as suspicious (RADS 4A and 4B criteria), which it can potentially prevent the need for unnecessary biopsies and/or additional dedicated imaging. We reclassified/downgraded suspicious cases from category 4 to 3 (probably benign) based on the probability of being benign as determined by M_NIS_. Consequently, the downgraded patients would undergo six months of low dose CT as per recommendations, thus potentially avoiding biopsy or reducing radiation exposure from short term follow up CT/PET-CT. Authors in [53] also showed that CT extracted features can improve the performance of lung-RADS. Our study is different from the work presented in [53]. While we used automatically extracted NIS features from baseline screening scans, the study in [53] used 24 handcrafted radiological image traits, such as vessel attachment, attenuation, air bronchogram, fissure attachment, pleural attachment, and nodules in the primary tumor lobe. They also benefited from baseline and two additional follow-up scans. Their model yielded an AUC of 0.72 for the best handcrafted feature on baseline scans. They also achieved an AUC of 0.74 with a combined model of semantic features and lung-RADS on baseline scans, in contrast the NIS classifier alone had an AUC = 0.77 which with the combination of other intra- and peri-tumoral texture features increased of 0.84. Further, we believe our NIS feature approach to more robust since the semantic scoring approach used in [53] involves manual feature extraction and hence is more subjective. Additionally, in contrast to our study, the training and test cohorts were relatively small in [53].

Limitations of this study include the retrospective design of our cohort, which was restricted to only adenocarcinomas and granulomas. However, it is worth noting that this is still one of the most challenging problems in lung nodule interpretations on CT scans and hence an extremely important clinical dilemma, especially in the Ohio River Valley and the upper Midwest region of the United States [54,55]. Although most benign conditions do not present with high FDG avidity on PET scan and thus do not present as much of a diagnostic dilemma as granulomas. Nonetheless, incorporating a broader range of benign conditions and including squamous cell cancers may expand the utility/applicability of our radiomic model. Multiple groups have also highlighted the importance of qualitative semantic features for nodule characterization, such as nodule location, cavitation, and calcification [45,46]. Hence, another future research avenue might involve integrating these radiologist-crafted features to analyze their importance in our cohort.

## 5. Conclusions

In conclusion, we introduced a new radiomics approach that demonstrates the utility of NIS features pertaining to differential texture transition along nodule interface on non-contrast chest CT imaging to discriminate adenocarcinomas from granulomas. Incorporating NIS features with intra-nodular texture improved the predictive ability of the classifier to distinguish adenocarcinomas from granulomas. Combining NIS with Lung-RADS has the potential to alter patient management by significantly decreasing unnecessary biopsies/follow up imaging.

## Figures and Tables

**Table 1:** Classification results (AUCs) of M_NIS_ and other classifiers trained on S_t_ and validated on S_v_ and S_iv_.

Dataset	M_NIS_	M_Intra-Tex_	M_Peri-Tex_	M_Shape_	M_All_
S_t_	0.83 ± 0.04	0.84 ± 0.04	0.82 ± 0.05	0.65 ± 0.05	0.91 ± 0.03
S_v_	0.77	0.73	0.71	0.64	0.80
S_iv_	0.71	0.62	0.63	0.66	0.70

**Table 2:** The effect of M_NIS_ benignity threshold on downgrading cases that were initially deemed as suspicious by lung-RADS alone.

Suspicious Cases	M_NIS_ Benignity	Downgraded Cases	Downgrade Ratio	TP	FP	NIS_Benefit
135	0.58	62	46%	46	16	0.74
135	0.68	60	44%	45	15	0.75
135	0.88	51	38%	39	12	0.76
135	0.98	36	27%	30	6	0.83

**Figure A1:**
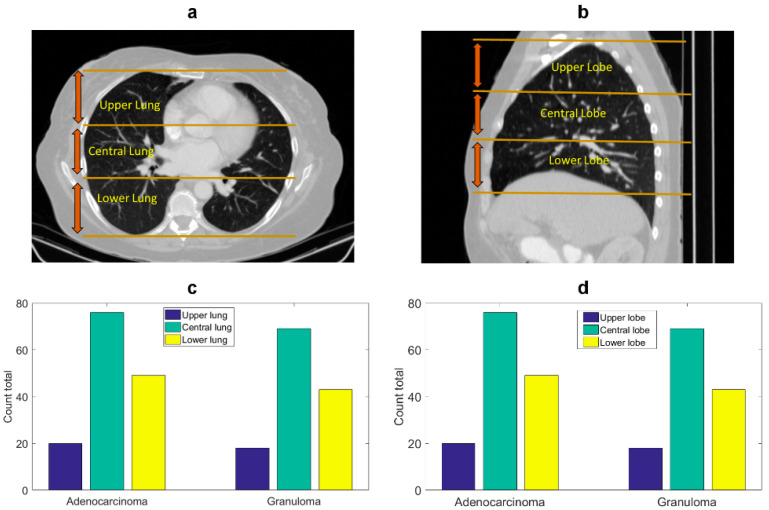
Association of nodule location and diagnostic class. Axial (**a**) and sagittal (**b**) plane CT images with each lung divided into upper, central and lower lung zone. Bar diagrams showing the distribution of the adenocarcinomas and granulomas based on their location in different zone on axial plane (**c**), and sagittal plane (**d**).

**Figure A2:**
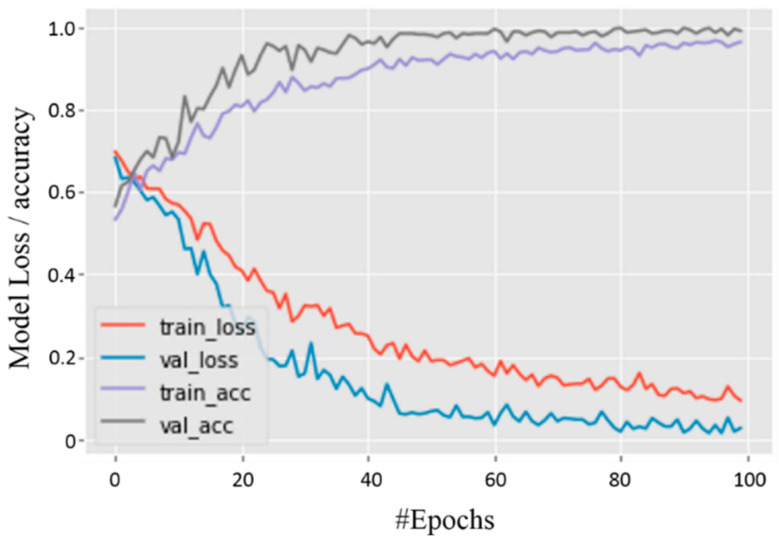
Training and Validation metrics across 100 epochs for the CNN. This plot shows that the model loss decreases over the epochs, and approaches zero at 100 epoch mark. Similarly, model accuracy approaches 1 as number of epochs increases, and plateaus at 80 epochs.

## Data Availability

Data is not publicly available due to IRB committee decision to keep the data private.

## Appendix A

### Appendix A.1. Extracted Features

**NIS features:**

*a. Shell Definition*

Let $\Gamma =\left\{1,\ldots ,H\right\}\times \left\{1,\ldots ,W\right\}\times \left\{1,\ldots ,D\right\}$ be a three-dimensional image lattice and v be the binary volume of a nodule defined as $v\;:\;\Gamma \;\to \;\left\{0,\;1\right\}$. The nodules volume is partitioned into *k* shells such that $v=\left\{s_{1},\;\ldots ,\;s_{k}\right\},\;s_{i-1}\subset \;s_{i}$ and $\cup_{i=1}^{k}s_{i}=v$. Each shell ($s_{i}$) is consisted of *n* slices (layers) $s_{i}=\left\{l_{1},\;\ldots ,\;l_{n}\right\}$ and each 2D slice ($l_{i}$) consisted of boundary pixels $l_{i}=\left\{p_{1},\;\ldots ,p_{j}\right\}$. The slope of normal line at a boundary pixel $p_{i}=\left(x,\;y\right)$, is computed using the co-ordinates of two adjacent pixels of it. As shown in Figure 2, the normal line at a boundary pixel $p_{i}$ is then divided into foreground (f) and background (b) pixels.

*b. NIS Features Definition*

The average gradient difference of every $p_{i}$ is then computed based on gradient values over f and b via:

(A1) $$\;dG_{p_{i}}=\frac{1}{Q}\sum_{q=1}^{Q}\frac{\nabla f_{q}-\nabla b_{q}}{2q}$$

where, $Q=\frac{R}{2}-1$ and R is the number of pixels sampled over the normal line of pixel $p_{i}$ and $\nabla f_{q}$, $\nabla b_{q}$ are the gradient magnitude values of foreground and background pixels along the normal line. Accordingly, the intensity difference profile $\;dI_{p_{i}}$ at pixel $p_{i}$ is calculated based on Equation (1) by plugging the intensity instead of gradient values. In addition to $\;dG_{p_{i}}$ and $\;dI_{p_{i}}$, the average gradient sharpness at pixel *pi* is defined as:

(A2) $$\;aG_{p_{i}}=\frac{1}{R}\overset{R}{\underset{r=1}{\sum }}M_{r}$$

where, $M_{r}\;$ is the gradient magnitude value of the *r* th sample over the normal line. Similarly, the entropy of the gradient magnitudes over the normal line of $p_{i}\;$ is calculated via:

(A3) $$\;\varepsilon_{p_{i}}=\overset{R}{\underset{r=1}{\sum }}M_{r}\log_{2}M_{r}$$

Finally, for each shell s ∈ v we calculated the mean, standard deviation, minimum and maximum of the $dG_{p_{i}}$, $\;dI_{p_{i}}$, $\;aG_{p_{i}}$, $\;\varepsilon_{p_{i}}$ according to the constituent border pixels p ∈ l of the 2D slices l ∈ s.

In the inner shell (*Sh_i_)* which corresponds to the intra-tumoral region, the average grayscale intensity profile and the first order derivative of intensity profiles over *l_p_* were found to be among the most stable and discriminating features. For the middle shell (*Sh_m_)* representing the boundary of the nodule, the standard deviation of grayscale intensity profile and derivative grayscale intensity profiles, as well as the average interface sharpness were found to be the most stable and discriminating features. Similarly, for the outer shell (*Sh_o_)*, the standard deviation of grayscale intensity profiles and the entropy of derivative grayscale profiles over *l_p_* were found to be most informative and stable.

**Shape features:**

Table A1 summarizes the extracted shape features from nodules.

**Table A1:** List of extracted shape features from CT.

Shape Features	Description
Size	Including Width, Height, Depth of bounding box
Area	from 2D slices of each nodule
Perimeter	from 2D slices of each nodule
Eccentricity	foci of the ellipse and to major axis length
Extend	ratio of pixels in the region to pixels in the total bounding box
Compactness	ratio of the perimeter squared to the product of 4π and area
Radial distance	distances from center of each slice to contour points
Roughness	perimeter of slices divided by convex perimeter
Elongation	from major and minor axis
Convexity	from convex hull
Equivalent Diameter	Diameter of circle with same area of slices
Sphericity	3D compactness

**Textural and peri-nodular features:**

The Table A2 introduces the textural feature categories that extracted from both nodular and peri-nodular regions.

**Table A2:** Extracted textural feature categories and their description.

Feature Category	Descriptor	Intuitive Description
Haralick features (Repeated occurrence of grey level configuration in the texture represented via the grey-level co-occurrence matrix (GLCM), which varies rapidly with distance in fine textures and slowly in large textures)	Inverse Difference Moment (IDM)	IDM is a reflection of the presence or absence of uniformity, and hence is a measure of local regions of homogeneity High IDM: Higher presence of locally uniform windows in GLCM Low IDM: Higher presence of locally heterogeneous windows in GLCM
	Correlation	Quantifies the linear patterns in an image based on the distance parameter.
	Sum Entropy	Measure of GLCM relationship to distribution of intensity with respect to entropy. Entropy is the measure of disorder.
	Sum Variance	Measure of GLCM relationship to distribution of intensity with respect to variance. High sum variance: greater standard deviation of sum average. Low sum variance: low standard deviation of sum average
Laws features	E5, L5, S5, R5 (combination in both *X* and *Y* directions)	E-Edges L-Level S-Spots R-Ripples
Gabor	Quantifies response to a given Gabor filter at a specific frequency and orientation	These filters comprise of various scales and orientations to locally characterize intensity variations

### Appendix A.2. Statistical Analysis between Patient and CT Specific Parameters with Histopathologic Diagnosis of the Nodule

Statistical significance test results between patient and CT parameters with clinical outcome for both the S_t_ and S_v_ cohorts is shown in Table A3. The presence of a statistically significant difference was indicated by *p* < 0.01. ‘Smoking status’ and ‘Age’ were the only patient parameters that were found to be significantly different between adenocarcinomas and granulomas in S_t_. While in S_v_ ‘Age’ was found to be significantly different and no significant differences were identified between remaining parameters of the adenocarcinomas and granulomas in S_t_ or S_v_.

**Table A3:** Details of the distribution of the granulomas and adenocarcinomas within the training and validation sets. *p*-values indicate the statistical significance between patients parameters and disease outcome for both training and validation cohorts. The *p*-values were computed using Students t test for continuous variable and Fishers exact test for categorical data.

Parameters	S_t_			S_v_		
	Adenocarcinoma	Granuloma	*p*-Value	Adenocarcinoma	Granuloma	*p*-Value
**Total Population**	**145**			**145**		
Subpopulation	73	72		73	72	
**Nodule Size (mm)**			**0.42**			**0.011**
Mean	13.33	11.15		11.91	12.19	
Std Deviation	6.65	4.45		4.36	6.48	
**Gender**			**0.31**			**0.50**
Male	27	33		31	35	
Female	46	39		42	37	
**Age (in years)**			**<0.01**			**<0.01**
Mean	73.87	62.85		72.08	61.31	
Std Deviation	10.34	14.2		10.7	12.54	
**Smoking Status**			**<0.01**			**0.05**
Yes	53	17		43	25	
No	2	20		8	13	
Not available	18	35		22	34	
**Ethnicity**			**0.82**			**0.68**
Caucasian	41	38		43	51	
African American	12	12		13	19	
Not available	20	22		17	2	

### Appendix A.3. Association of the Nodule Location and Diagnostic Class

To determine the association of a nodule’s spatial location with its corresponding diagnostic class, we captured whether a nodule was located in the upper, lower or central lung zones in axial and sagittal planes (see Figure A1). A frequency plot for the nodule position for both adenocarcinomas and granulomas is shown in Figure A1c,d. Performing $\chi^{2}$ test it was found that there was no significant association between the nodule location (in both axial and sagittal planes) and its diagnostic class. The corresponding *p*-values are shown in the Table A4.

**Table A4:** The *p*-values corresponding to the $\chi^{2}$ test between the diagnostic class of a nodule and its relative position in axial and sagittal planes. *p*-value < 0.01 was indicating the presence of a significant association.

Nodule Position vs. Nodule Class	2D				3D			
	Training Set		Validation Set		Training Set		Validation Set	
*p*-value	Left lung 0.05	Right lung 0.92	Left lung 0.49	Right lung 0.64	Left lung 0.06	Right lung 0.38	Left lung 0.75	Right lung 0.015

### Appendix A.4. Deep Learning Performance across the Training Runs

The LeNet and 3D CNN architectures comprised two sets of convolutional, rectified linear unit (ReLU) activation, and pooling layers, followed by a fully-connected layer, activation, another fully-connected, and finally a softmax classifier. The CNN models were trained with more than 100 epochs, after which the weights were locked down for testing. Weights learned from the training phase were then used on S_v_ to classify nodules. The learned weights were then evaluated on S_v_, and the predicted probabilities were utilized to generate the ROC curve. To extract deep features using a 2D CNN, 2D image patches with a receptive field size of 80 × 80 pixels were cropped at the center of the nodule across all slices, these were then inputted to a 2D CNN. The 3D model also used 3D patches with a receptive field size of 50 pixels in the XY plane and 10 slices in the Z plane (50 × 50 × 10). The performance metric across the training runs are shown in Figure A2. The learned weights were then used on the independent validation set of 145 studies, and the predicted probabilities were utilized to generate the receiver operating characteristic curve.

### Appendix A.5. The Accuracy of the Radiomic Models

The accuracy of the M_NIS_ and other radiomic models is shown in Table A5.

**Table A5:** The accuracy of the M_NIS_ and other radiomic models in distinguishing adenocarcinomas from granulomas on S_t_, S_v_ and S_iv_ datasets.

Dataset/Model	M_NIS_	M_Intra-Tex_	M_Peri-Tex_	M_shape_	M_All_
S_t_	0.73 ± 0.04	0.79 ± 0.03	0.78 ± 0.05	0.63 ± 0.03	0.82 ± 0.05
S_v_	0.65	0.68	0.65	0.61	0.71
S_iv_	0.80	0.42	0.85	0.66	0.73
